# Deficits in odor discrimination versus odor identification in patients with schizophrenia and negative correlations with GABAergic and DNA methyltransferase mRNAs in lymphocytes

**DOI:** 10.3389/fpsyt.2023.1115399

**Published:** 2023-03-28

**Authors:** Robert C. Smith, Henry Sershen, Mary Youssef, Abel Lajtha, Hua Jin, Mumei Zhang, Anmei Chen, Alessandro Guidotti, John M. Davis

**Affiliations:** ^1^Nathan Kline Institute for Psychiatric Research, Orangeburg, NY, United States; ^2^Department of Psychiatry, NYU Grossman School of Medicine, New York, NY, United States; ^3^Columbia University Mailman School of Public Health, New York, NY, United States; ^4^Department of Psychiatry and VA San Diego Healthcare System, University of California San Diego, San Diego, CA, United States; ^5^Department of Psychiatry, Psychiatric Institute University of Illinois, Chicago, IL, United States

**Keywords:** olfactory deficits, schizophrenia, GABAergic mRNA, odor discrimination, MATRICS battery

## Abstract

**Introduction:**

People with schizophrenia have been reported to show deficits in tests of olfactory function. DNA methylation and GABAergic input have been implicated in biochemical processes controlling odor in animal studies, but this has not been investigated in human studies.

**Methods:**

In a study of measures of DNA methylation and GABAergic mRNAs in lymphocytes, we also measured odor identification and discrimination with the Sniffin’ Sticks battery in 58 patients with chronic schizophrenia (CSZ) and 48 controls. mRNAs in lymphocytes were assessed by qPCR using TaqManTM probes. Cognition was assessed by the MATRICS battery (Measurement and Treatment Research to Improve Cognition in Schizophrenia) in CSZ and controls, and symptoms in CSZ were assessed by PANSS scale (Positive and Negative Symptom Scale). The relationships of odor deficits with mRNA, cognition, and symptoms were explored by correlation analysis. Variables which significantly differentiated CSZ from controls were explored by logistic regression.

**Results:**

Overall, CSZ showed significantly (P≤.001) lower scores on odor discrimination compared to controls, with a moderate effect size, but no difference in odor identification. Deficits in odor discrimination, which has not been standardly assessed in many prior studies, strongly differentiated CSZ from controls. In logistic regression analysis, odor discrimination, but not odor identification, was a significant variable predicting schizophrenia versus control class membership. This is the first study to report relationship between odor deficits and DNA methylation and GABAergic mRNAs in blood cells of human subjects. There were negative correlations of odor identification with DNA methylation enzymes mRNAs and significant negative correlations with odor discrimination and GABAergic mRNAs. Lower odor scores were significantly associated with lower cognitive scores on the MATRICS battery in CSZ but not control subjects. In CSZ, lower odor scores were significantly associated with negative symptom scores, while higher odor identification scores were associated with PANNS Excitement factor.

**Discussion:**

Odor discrimination was a more powerful variable than odor identification in discriminating CSZ from controls and should be used more regularly as an odor measure in studies of schizophrenia. The substantive meaning of the negative correlations of odor discrimination and GABAergic mRNA variables in peripheral lymphocytes of CSZ needs more investigation and comparison with results in neural tissue.

## Introduction

Numerous studies have reported deficits in olfactory function in people with schizophrenia compared to controls as summarized in several reviews (1–4). These have included deficits in odor identification, threshold sensitivity, and hedonistic valence, with somewhat less attention to odor discrimination. These deficits have been found in patients with chronic schizophrenia (CSZ), in addition to first-episode schizophrenia, and some studies have reported odor deficits in family members of patients with schizophrenia and subjects who are judged to be at clinical high risk for developing schizophrenia (1, 3, 5, 6). Odor deficits have been related to symptoms and cognitive deficits in schizophrenia.

Studies in animals indicate that DNA methyltransferase activation or silencing is involved in the development of the olfactory nervous system and neural processing in odor learning and discrimination (7). Other studies indicate that GABA modulation is important for appropriate odor perception involving effects on synaptic transmission in the olfactory bulb and controlling network oscillations (8–10). Basal forebrain GABAergic neurons, targeting local inhibitory neurons in the olfactory bulb, can influence the temporal and spatial dynamics of odor coding in the olfactory bulb; disruption of GABA inhibition impairs discrimination of similar odors (10). There has been limited investigation on whether these biochemical modulations may be involved in odor functioning in humans, although one study suggests that DNA methylation markers measured in white blood cells are involved with regulation of genes in the olfactory pathway (11). Furthermore, abnormalities in the GABAergic system have been reported prominently in schizophrenia. This decrease in GABAergic function in schizophrenia may be caused by the epigenetic silencing of glutamic acid decarboxylase_67_ (GAD_67_) expression due to both increase DNA methyltransferase (DNMT) and lower levels of histone deacetylase2 (HDAC2 activity) in dorsolateral prefrontal cortex observed in postmortem brains of patients with schizophrenia, as well as some similar findings in peripheral lymphocytes (12–17). Postmortem brains of patients with schizophrenia also show a decrease in synaptic spines in GABA-related neurons; a finding consistent with the decrease in gray matter found in schizophrenia and associated the cognitive deficits (18–20).

In a study of methylation-related mRNA differences in CSZ and controls in peripheral blood lymphocytes (PBLs), we also assessed odor identification and discrimination in addition to psychopathology and cognitive deficits. We report on the extent of odor deficits in this sample and their relationship to biochemical markers in lymphocytes and to the clinical-related variables. We assess the strength of odor discrimination versus odor identification in characterizing patients with chronic schizophrenia.

## Methods

### Subjects and study design

The design of the original study from which data for this paper was drawn is described in detail in our previous publication (15). Subjects were enrolled in this study and samples collected between 2013 and 2017. Subjects included in the current report were 58 CSZ patients treated with antipsychotic medication, and 48 controls (Table 1). CSZ were recruited from the Nathan Kline Institute (NKI) or its associated state hospital, outpatient clinic, and residences. Control subjects were recruited from the NKI research clinic, or from the local community through advertisements. CSZ (34 outpatients, 24 inpatients) subjects had a long history of illness with several hospitalizations and/or years of outpatient clinic treatment in multiple institutions; available chart records did not provide reliable data to specify precise years of illness or treatment duration. Diagnoses of schizophrenia was made by review of hospital records, using checklists for DSM IV and later DSM V, and supplemented by SCID diagnostic interviews when these were available from other studies. Controls were subjects who never met criteria for schizophrenia, bipolar disorder, major depressive disorder, schizophreniform disorder, or brief or drug-induced psychosis, and were not currently treated with antipsychotic or antidepressant medication (see supplement for additional details of selection criteria). None of the control subjects were treated with a psychotropic drug for a psychiatric diagnosis. Subjects signed informed consent forms for participation in a protocol approved by the IRB of the Nathan Kline Institute for Psychiatric Research.

**Table 1:** Characteristics of subjects.

Characteristic	Schizophrenia–CSZ	Controls	Test
	(*n* = 58)	(*n* = 48)	
Age (m)	44.7 ± 9.6	35.6 ± 10.9	T = 4.57, df = 104, *p* = <0.001
Sex (M/F) (n)	50/8	32/16	*Χ*^2^ = 5.73, df = 1 *p* = 0.017
Race (W/B/H/A) (n)	16/37/3/2	13/31/2/2	FET = 1.000
Cigarette smoker (Y/N) (n)	36/22	9/39	*Χ*^2^ = 20.17df = 1, *p* < 0.001
Cigarette smoked/wk. (m)	36.2 ± 45.0	11.8 ± 27.8	*T* = 3.26, df = 104, *p* = 0.001
Handedness (right/left)	54/4	45/3	*Χ*^2^ = 0.02, df = 1, *p* = 0.89
Antipsychotic treatment (lst Gen/2nd Gen/combined (n)	4/36/18	NR	
On Clozapine (Y/N) (n)	27/31	NR	
On Antidepressant (Y/N/) (n)	6/52	0/48	
On Mood stabilizer (Y/N) (n)	23/35	0/48	
On Valproate (Y/N) (n)	9/49	0/48	
On Benzodiazepine (Y/N) (n)	22/38	0/48	
PANSS total (m)	71.6 ± 15.1	NR	
MATRICS overall composite (m)	21.45 ± 12.57	41.53 ± 9.76	*F* = 8.89, df = 100, *p* < 0.001
Urine Tox THC (P/N) (n)	0/24	4/32	FET = 0.13

NR, not relevant; (*n*), number of subjects; m, Mean ± S.D.; (Y/N), Yes/No; (P/N), Positive/Negative; (M/F), male/female, Race/Ethnicity: W, Caucasian; B, Black or African American; H, Hispanic; A, Asian. Antipsychotic type: lst Generation antipsychotic, 2nd generation antipsychotic, combined lst and 2nd Generation antipsychotic. Statistical tests: *Χ*^2^, chi-square; FET, Fishers’ exact test; *T*, *t*-test. We did not collect data on education level of our subjects. Cigarettes smoked per week is the subject’s response estimate of how many cigarettes they have smoked per week in the last few weeks.

### Measurement of odor deficits

Odor identification and odor discrimination were assessed with Sniffin’ Sticks pen like odor test kits; the properties of which are described in detail by Hummel and associates (21). We did not measure odor threshold. For odor identification assessment, 16 pens with different odors were presented to the subject at approximately 2 cm from both nostrils for 3 s. The subject was then presented with a card listing 4 possible odors from that pen and instructed to select the odor just smelled. If the subject identified the correct odor, the subject was given a score of 1 for that trial; otherwise, a score of 0. The maximum score was 16. For the odor discrimination assessment, 16 sets of three odor pens were presented, two of the same odors and one of a different odor. Each odor stick was presented for 3 s, and there was a 30 s interval between presentation of the triplets. The subject had to identify the odor (first, second or third) which was different. For the discrimination task the subjects wore a mask over their eyes. For each correct choice the subject received a score of 1 and for each incorrect choice a score of 0. The maximum score was 16.

### Clinical assessments

Psychopathology in CSZ was assessed with the Positive and Negative Symptom Scale (PANSS) (22) by interview by trained research psychiatrists, or research assistants who had achieved at least an ICC of 0.80 with total PANSS score rating agreement with psychiatrist’s ratings. We analyzed total PANSS score and examined results of 5 factors derived from the PANNS scale established through previous factor analysis of the PANSS items (23, 24).

Cognitive Status was assessed by the Measurement and Treatment to Improve Cognition in Schizophrenia (MATRICS) battery (25) by raters who were trained in the battery’s procedures.

### RNA extraction and gene expression assay

RNA was isolated from blood lymphocyte samples and measures of gene expression assayed for genes of interest. Lymphocyte collection and qPCR assays are described fully in our previous publication (15). Subject’s blood was collected in ~4 × 10 ml EDTA tubes, put in an ice bucket, and rapidly processed. Lymphocyte (peripheral blood lymphocytes (PBL)) were extracted by Ficoll gradient procedures and pellets frozen as described previously. RNA was extracted from lymphocyte pellets with a TRIzol procedure. First strand cDNA was prepared using the Invitrogen SuperScript VILO cDNA Synthesis kit; up to 2.5 μg RNA measured with NanoDrop Lite (Thermo Scientific) was reacted with reagent mix, incubated at 42^o^ C for 60 min, and terminated at 85^o^ C for 5 min. The sample was frozen (−80^o^ C) until assayed. For qPCR, TaqMan Universal PCR Master Mix was used for target amplification using the cDNA template and using primer/probes from the TaqMan Gene Expression Assay mix (see Table 2 for probes). Samples were assayed in triplicate, normalized against β-actin as the housekeeping gene, and ddCt = 2^(−dt) values calculated.

**Table 2:** Gene symbols and TaqMan primers.

Primer probe gene symbol	Taqman assay gene expression	Gene name
ACTB	Hs01060665_g1	Actin beta
DNMT1 (original, OR)	Hs00154749_m1	DNA (cytosine-5-)-methyltransferase 1 Transcript variant 1 (DNMT1b), 2 (DNMT1a), and variants 3,4
DNMT1 CP (custom probe)	AIFAT65	DNA (cytosine-5-)-methyltransferase 1\u00B0F: 5′-CGTCTAGAAAACGGGAACCAAGCAAG-3′ R: 5′-TCTAATCCCAGTTACTTGGGAGGCTG-3′
DNMT3A	Hs01027166_m1	DNA methyltransferase 3 alpha
GAD1	Hs01065893_m1	glutamate decarboxylase 1 (full length, multiple transcripts)
GAD1 (variant GAD67)	Hs01065886_m1	glutamate decarboxylase 1 (truncated form GAD67)
GAD1 (variant GAD25)	Hs00247564_m1	glutamate decarboxylase 1 (truncated form GAD25)

The differing portions of the gene covered in the original and these duplicate TaqMan probes for the same main gene are further specified in the information under the Gene Name column in the table. The GAD1 full length TaqMan probe contains the transcripts for the variants GAD67, GAD25, and multiple other transcripts.

## Statistical analysis

We analyzed our data using SPSS 25 and SAS 9.4. Statistical significance was set at *p* < 0.05, 2 -tailed and trend level at *p* < 0.10. We tested variables for normality using SPSS Explore and where the distributions markedly deviated from normality, we attempted transforms (log, Ln, square root) to normalize the distributions. Where they remained markedly non-normal we used non-parametric tests. To analyze differences between CSZ and controls we used a complex analysis of variance with age and cigarette smoking as covariates and sex as factor; we also utilized multiple regression equation with forward step-down inclusion procedures to test-which background variables contributed significantly to prediction of odor scores. To assess the relationship of odor scores to biochemical, cognitive and psychopathology variables we used parametric (r) and non-parametric (rho) correlations. To test the effects of sex on the correlation between MATRICS variables and odor deficits, we used multiple regression analysis to assess the sex effect in schizophrenia patients, with the outcome as either the odor identification or odor discrimination. Three variables were included in the model: cognitive measures, an interaction term constructed from its product with sex, and sex alone. Similar multiple regression models were used to assess whether there were any significant effects of sex on the correlations of odor identification or discrimination with the mRNA variables. For each multiple regression model, we included the mRNA variables, sex, and their interaction terms as the predictors, and either odor identification or odor discrimination as the outcome. In these models, we mainly focused on examining the effects of the interaction terms. To assess which variables significantly differentiated controls from patients with schizophrenia, we used logistic regression analysis. In the main analysis, variables used in the models include odor identification score, odor discrimination score, MATRICS domain variables, age, sex, and two different smoking variables, smoker/non-smoker status and the number of cigarettes smoked per week. In the additional analysis, the DNMT and GABAergic mRNA variables are added to the models separately, to examine the effects of these variables in predicting schizophrenia and control membership. In this paper, we have also performed causal mediation analysis using the model-based approach with an R package named Mediation. We have fitted two ordinary least squares regression models for each analysis: the mediator model and the outcome model. To examine the mediation effect of odor discrimination of diagnosis onto odor identification, we have fitted the models with potential mediator odor discrimination, exposure diagnosis group, and outcome odor identification. To examine whether the selected MATRICS cognitive measures or the selected mRNA variables mediate the effect of diagnosis onto odor identification or odor discrimination, we have fitted the models with the potential mediator as the selected MATRICS cognitive measures variables, or the selected mRNA variables, exposure diagnosis group and outcome as odor discrimination or odor identification. All models have controlled for covariates age, sex, and the number of cigarettes smoked. The mediate function was used in both analyses to estimate the average causal mediation effect (ACME) and the average direct effect (ADE) of the fitted models. (Further details of the statistical methods can be found in the Supplementary data).

## Results

### Background characteristics of sample

Table 1 shows the characteristics of our sample. This was a predominately male sample with a smaller number of female participants. Mean age of CSZ was older than the controls and CSZ had a significantly greater number of cigarette smokers. All CSZ were treated with antipsychotics and CSZ showed moderate symptomatology based on PANSS scores with a wide range (mean 72, range 32–102). CSZ had substantially lower cognitive scores on the MATRICS than controls. The CSZ patients had been ill for many years although we did not have precise data on years of illness and hospitalizations. The very low MATRICS scores of the CSZ subjects may be due to their long illness. There was no explicit data on education levels in many of the CSZ subjects’ charts and we did not record education level in our background variables.

There were no significant differences (by t-test) in background characteristics of CSZ or controls comparing those subjects who had mRNA data on DNMT1 or GAD1 versus subjects in the entire sample (see Supplementary Tables S1, S2).

### Odor identification and discrimination in CSZ versus controls

**Figure 1:**
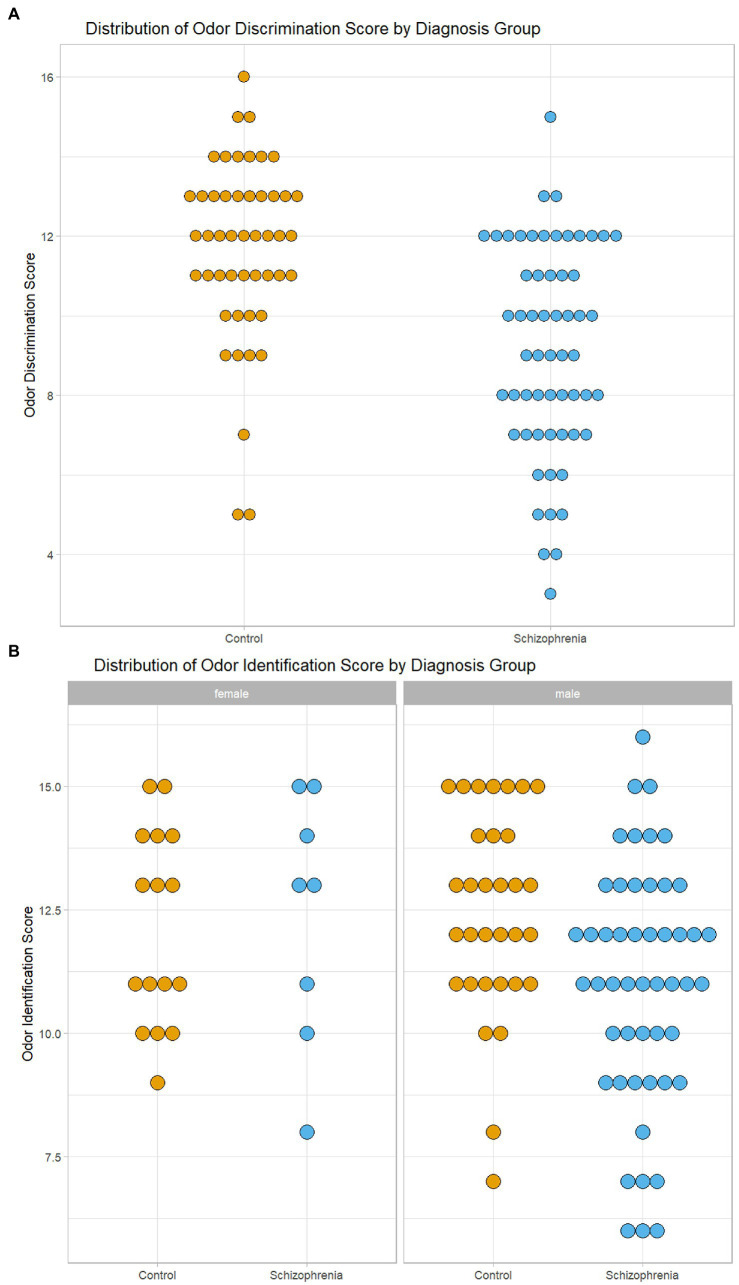
Graphical Display of Distribution of Odor Scores in Subjects with Schizophrenia and Controls. **(A)** Distribution of Odor Discrimination Scores in Subjects with Schizophrenia and Controls. **(B)** Distribution of Odor Identification Scores by Sex in Subjects with Schizophrenia and Controls.

Because there were differences in age, sex and cigarette smoking between the CSZ and control samples, we analyzed for significant differences in odor identification and odor discrimination between CSZ and controls in an analysis of variance using these variables as factors or covariates. As shown in Table 3, there was no significant difference between CSZ and controls in the adjusted means of odor identification but a highly significant difference in odor discrimination with a relatively large effect size. Figure 1A, which shows the distribution of raw odor discrimination scores in the CSZ and control groups, presents this graphically. However, for odor identification when we analyzed males and females separately, male CSZ showed significantly lower scores in odor identification compared to controls (*F* = 4.778 df = 1,78 *p* = 0.032; CSZ 11.07 ± 0.34 Control 12.33 ± 0.43), whereas females showed no difference between the two groups (*F* = 0.005 df = 1,20 *p* = 0.943; CSZ 12.26 ± 0.77 Control 12.14 ± 0.52). For odor discrimination, however both males and females showed a diagnoses effect with CSZ having lower scores than controls (males *F* = 11.203, df = 1,78, *p* = 0.001, females *F* = 7.922 df = 1,20, *p* = 0.011). Figure 1B presents graphically the sex effects for odor identification. However, overall age sex and smoking status were not strong effects as predictors of differences in these two olfactory functions. There were no significant correlations between number of cigarettes smoked per week and odor identification or discrimination in the whole sample or the CSZ group. In a multiple regression equation with forward step-down inclusion procedures, only diagnosis yielded a significant β (odor discrimination *β* = −0.454, *t* = 5.19, *p* < 0.001; odor identification *β* = −0.246, *t* = 2.59 *p* = 0.011), and the contribution of age, cigarette smoking status and sex were not significant predictors of odor identification or discrimination (P’s all >0.17), There were no significant differences in odor identification or discrimination scores in CSZ by type of antipsychotic (first generation, second generation, combined first or second generation) (ANOVA: odor identification *F* = 0.234, df = 2,58, *p* = 0.792, odor discrimination *F* = 0.630, df = 2,58,*p* = 0.536). However, CSZ treated with clozapine had significantly lower odor discrimination scores than CSZ not treated with clozapine (*t* = 2.34, df = 55, *p* = 0.023), although both groups were lower than controls. There was no significant difference in odor identification scores by clozapine treatment (*t* = 1.54, df = 56, *p* = 0.106).

**Table 3:** Differences in odor identification and discrimination between controls and patients with Chronic Schizophrenia (CSZ).

Odor measure	Controls *n* = 48	Schizophrenia (CSZ) *n* = 58	Analysis of variance	Effect size
Sum of odor identification	12.18 ± 0.37	11.78 ± 0.44	*F* = 0.433, df = 1,100 *p* = 0.512	−0.14 (−0.52 − +0.25)
Sum of odor discrimination	11.80 ± 0.41	9.38 ± 0.48	*F* = 13.708, df = 1,100, p < 0.001	−0.73 (−1.12- -0.39)

Each number is adjusted mean ± s.e.m. from analysis of variance with age and number of cigarettes smoked /week as covariates and sex as factor. Effect Size is Cohen’s d with (95% confidence interval).

Similar results were obtained for the full sample and the reduced number of subjects who had values for DNMT1 or GAD1 measured. In multiple regression equations, there were no significant interaction term effects between the characteristics of full sample versus reduced sample and the diagnostic effect (schizophrenia versus controls) on odor discrimination or odor identification (DNMT1 odor identification *F* = 2.70 df = 1, p = 0.10, odor discrimination *F* = 2.10, df = 1, *p* = 0.15; GAD1 odor identification *F* = 2.11 df = 1, p = 0.15, odor discrimination *F* = 0.49, df = 1, *p* = 0.48).

### Correlations of odor scores with biochemical and behavioral measures

**Figure 2:**
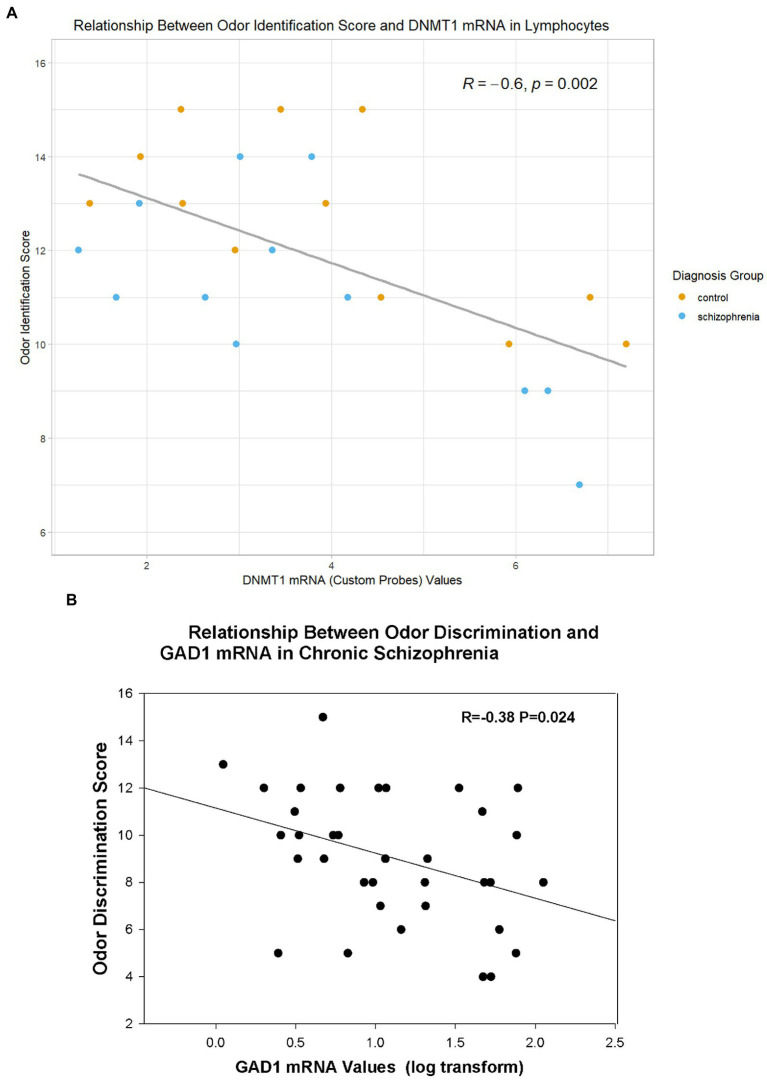
Relationship of Odor Variables to Selective mRNA Expression in Lymphocytes. **(A)** Relationship of DNMT1 (Custom Probe) to Odor Identification (in all subjects with DNMT1 CP values). **(B)** Relationship of GAD1 mRNA to Odor Discrimination in Subjects With Schizophrenia. R=Pearson correlation coefficient.

In the combined sample of CSZ and control subjects, DNMT1 mRNA levels in PBLs correlated negatively with odor identification scores (Figure 2A, Table 4), and GABAergic mRNA’s levels (GAD1, GAD25, GAD67) in PBLs correlated negatively with odor discrimination scores (Figure 2B, Table 5). However, the significant correlation of odor identification with a variant of DNMT1 (DNMT1 CP) was based only on a small sub-sample of subjects for which we assayed with this mRNA probe. There were no significant correlations with DNMT3A. In CSZ the GABAergic correlations tended to be more highly negative and statistically significant than in controls where the correlations were very small. In the CSZ subjects there was a trend (*p* < 0.10) for subjects with low odor discrimination scores ≤7 to have higher levels of GAD25 and GAD67 mRNA than subjects who had scores ≥12. Multiple regression analysis showed no sex effects on the correlations between mRNA variables and odor identification or discrimination (see Supplement for details of method).

**Table 4:** Correlations between odor identification scores and mRNA and cognitive measures.

Measure	Total sample	CSZ subjects	Control subjects
*mRNA levels in lymphocytes*			
DNMT1 OR (lg)	*r* = −0.291 *p* = 0.026, *n* = 58	*r* = −0.330 *p* = 0.087 *n* = 28	*r* = −0.210 *p* = 0.266 *n* = 30
DNMT1 CP	*r* = −0.603 *p* = 0.002, *n* = 24	*r* = −0.688 *p* = 0.013 *n* = 12	*r* = −0.688 *p* = 0.013 *n* = 12
*MATRICS cognitive measures*			
Speed of processing	*r* = 0.359 *p* < 0.001 *n* = 104	*r* = 0.304 *p* = 0.022 *n* = 57	r = 0.240 *p* = 0.104 *n* = 47
Attention vigilance	*r* = 0.205 *p* = 0.039 *n* = 102	*r* = 0.195 *p* = 0.153 *n* = 55	r = −0.066 *p* = 0.660 *n* = 47
Working memory	*r* = 0.291 *p* = 0.003 *n* = 104	*r* = 0.273 *p* = 0.040 *n* = 57	r = 0.057 *p* = 0.702 *n* = 47
Verbal learning	*r* = 0.190 *p* = 0.053 *n* = 104	*r* = 0.113 *p* = 0.402 *n* = 57	*r* = 0.032 *p* = 0.829 *n* = 47
Visual learning	*r* = 0.208 *p* = 0.034 *n* = 104	*r* = 0.160 *p* = 0.235 *n* = 57	*r* = 0.055 *p* = 0.713 *n* = 47
Reasoning/Problem solving	*r* = 0.259 *p* = 0.008 *n* = 104	*r* = 0.295 *p* = 0.026 *n* = 57	*r* = 0.082 *p* = 0.583 *n* = 47
Social cognition	*r* = 0.090 *p* = 0.364 *n* = 103	*r* = −0.052 *p* = 0.703, *n* = 56	*r* = −0.176 *p* = 0.236 *n* = 47
Overall composite score	*r* = 0.315 *p* = 0.001 *n* = 102	*r* = 0.300 *p* = 0.026 *n* = 55	*r* = 0.061 *p* = 0.684 *n* = 47

*r*, Pearson correlation coefficient. GABAergic mRNAs showed no significant correlations with odor identification scores. lg is log_10_ transformation of DNMT1 values which was used in the analysis because it provided a more normalized distribution of values for this variable.

**Table 5:** Correlations between odor discrimination scores and mRNA and cognitive measures.

Measure	Total sample	CSZ subjects	Control subjects
*mRNA levels in lymphocytes*			
GAD1(lg)	*r* = −0.392 *p* = 0.001, *n* = 65	*r* = −0.380 *p* = 0.024 *n* = 35	*r* = −0.081 *p* = 0.669 *n* = 30
GAD25 (lg)	*r* = −0.350 *p* = 0.003, *n* = 68	*r* = −0.424 *p* = 0.01 *n* = 36	*r* = −0.094 *p* = 0.609 *n* = 32
GAD67	rho = −0.323 *p* = 0.007 *n* = 68	rho = −0.408 *p* = 0.014 *n* = 36	rho = −0.106 *p* = 0.565 *n* = 32
*MATRICS cognitive measures*			
Speed of processing	*r* = 0.419 *p* < 0.001 *n* = 104	*r* = 0.223 *p* = 0.095 *n* = 57	*r* = 0.142 *p* = 0.340 *n* = 47
Attention vigilance	*r* = 0.373 *p* < 0.001 *n* = 102	*r* = 0.217 *p* = 0.111 *n* = 55	*r* = 0.135 *p* = 0.364 *n* = 47
Working memory	*r* = 0.480 *p* < 0.001 *n* = 104	*r* = 0.345 *p* = 0.009 *n* = 57	*r* = 0.226 *p* = 0.127 *n* = 47
Verbal learning	*r* = 0.421 *p* < 0.001 *n* = 104	*r* = 0.300 *p* = 0.023 *n* = 57	*r* = 0.147 *p* = 0.324 *n* = 47
Visual learning	*r* = 0.282 *p* = 0.004 *n* = 104	*r* = 0.166 *p* = 0.216 *n* = 57	*r* = 0.012 *p* = 0.934 *n* = 47
Reasoning/Problem solving	*r* = 0.261 *p* = 0.007 *n* = 104	*r* = 0.127 *p* = 0.345 *n* = 57	*r* = 0.099 *p* = 0.509 *n* = 47
Social cognition	*r* = 0.273 *p* = 0.005 *n* = 103	*r* = 0.101 *p* = 0.461 *n* = 56	*r* = −0.036 *p* = 0.812 *n* = 47
Overall composite score	*r* = 0.486 *p* < 0.001 *n* = 102	*r* = 0.328 *p* = 0.014 *n* = 55	*r* = 0.181 *p* = 0.225 *n* = 47

*r*, Pearson correlation coefficient; Rho, non-parametric correlation coefficient. DNMT1 or DNMT3A mRNAs were not significantly correlated with odor discrimination scores. (lg) is log_10_ transformation of variable which provided a more normal distribution of values.

Higher scores on odor identification and odor discrimination were correlated positively with some cognitive functions as measured by the MATRICS battery (Tables 4, 5). In the combined sample there were significant correlations between all the MATRICS measures and scores of odor identification and discrimination. However, further analysis showed that the significant correlations were found in the CSZ patients and not in the control subjects. The CSZ had modest (0.30–0.33) correlations with MATRICS overall composite score. In CSZ higher scores on odor identification correlated positively with MATRICS domains of speed of processing, working memory, and reasoning-problem solving, and higher scores on odor discrimination correlated positively with domain scores of working memory and verbal learning. However, in CSZ subjects the correlations of MATRICS variables with odor identification versus odor discrimination were not significantly different (P’s > 0.05) (see supplement for correlation comparison method). In CSZ subjects the correlations with MATRICS scores tended to be higher in the small sample (N = 8) of female CSZ than male CSZ, and for odor discrimination much larger and significant in the female CSZ sample (Table 6). Multiple regression analysis to assess the sex effect on the correlations between odor deficits and MATRICS scores in CSZ subjects showed that for odor discrimination there was a significant sex effect on the correlation of visual learning and social cognition domain scores, where female CSZ showed a significantly stronger correlation than male schizophrenia patients. There were no statistically significant sex effects on odor identification correlations with MATRICS variables (Table 6) (see Supplementary data for statistical methods.)

**Table 6:** Correlations between odor identification or odor discrimination scores and cognitive measures and interaction effects between sex and cognitive measures in subjects with Chronic Schizophrenia (CSZ).

	Odor identification			Odor discrimination		
MATRICS cognitive measure	Correlation male CSZ (*n* = 48–49)	Correlation female CSZ (*n* = 7–8)	Interactions with sex^a^	Correlation male CSZ (*n* = 48–49)	Correlation female CSZ (*n* = 7–8)	Interactions with sex^a^
Speed of processing	*r* = 0.301 *p* = 0.036	*r* = 0.600 *p* = 0.116	*F* = 1.09 *p* = 0.301	*r* = 0.194 *p* = 0.181	*r* = 0.561 *p* = 0.148	*F* = 1.22 *p* = 0.275
Attention Vigilance	*r* = 0.119 *p* = 0.420	*r* = 0.573 *p* = 0.178	*F* = 1.37 *p* = 0.247	*r* = 0.141 *p* = 0.340	*r* = 0.667 *p* = 0.102	*F* = 1.74 *p* = 0.193
Working Memory	*r* = 0.171 *p* = 0.239	*r* = 0.692 *p* = 0.057	*F* = 1.54 *p* = 0.220	*r* = 0.245 *p* = 0.089	*r* = 0.840 *p* = 0.009	*F* = 1.96 *p* = 0.167
Verbal Learning	*r* = 0.094 *p* = 0.521	*r* = 0.431 *p* = 0.286	*F* = 0.85 *p* = 0.362	*r* = 0.249 *p* = 0.085	*r* = 0.759 *p* = 0.029	*F* = 2.20 *p* = 0.144
Visual Learning	*r* = 0.094 *p* = 0.521	*r* = 0.324 *p* = 0.434	*F* = 0.36 *p* = 0.548	*r* = 0.039 *p* = 0.788	*r* = 0.838 *p* = 0.009	***F* = 4.25 *p* = 0.044**
Reasoning/Problem Solving	*r* = 0.264 *p* = 0.067	*r* = 0.338 *p* = 0.413	*F* = 0.10 *p* = 0.751	*r* = 0.135 *p* = 0.355	*r* = −0.011 *p* = 0.980	*F* = 0.10 *p* = 0.751
Social cognition	*r* = −0.060 *p* = 0.687	*r* = 0.321 *p* = 0.439	*F* = 0.92 *p* = 0.343	*r* = −0.054 *p* = 0.714	*r* = 0.796 *p* = 0.018	***F* = 4.63 *p* = 0.036**
Overall composite score	*r* = 0.212 *p* = 0.149	*r* = 0.704 *p* = 0.078	*F* = 1.59 *p* = 0.213	*r* = 0.222 *p* = 0.129	*r* = 0.931 *p* = 0.002	*F* = 3.24 *p* = 0.078

*r* = Pearson correlation coefficient. ^a^Represents the significance of additional effect of sex on the correlation with the cognitive variable in females compared to the cognitive variable in males. Statistic derived from multiple regression analysis to assess the sex effect in schizophrenia patients. Bolded values are statistically significant F at *P*< 0.05.

In CSZ subjects, odor discrimination had modest but significant correlations with PANSS Total and PANNS Negative and Depression factor scores, whereas odor identification score was correlated with PANSS Negative and Excitement factor scores but not PANSS Total score. There were no significant correlations with PANSS Positive factor. CSZ who had higher odor scores had lower negative symptoms (Table 7). However, those having higher odor identification scores had higher PANSS Excitement factor scores. Correlations tended to be higher in female than male CSZ, but there were no statistically significant sex effects on these correlations. The correlations with PANSS Depression factor were significantly different (*p* = 0.004) for odor discrimination versus odor identification (see Supplementary data for methods of correlation comparisons).

**Table 7:** Relationship of odor identification and discrimination to PANSS scores in patients with chronic schizophrenia, PANSS five factor analysis, and PANNS traditional summary scores.

PANSS score	Odor identification correlation	Odor discrimination correlation
PANSS total score	*r* = −0.111 *p* = 0.406	***r* =** −0**.313 *p* = 0.017**
*PANSS five factor scores*		
PANSS positive factor	*r* = 0.013 *p* = 0.921	*r* = −0.045 *p* = 0.738
PANSS negative factor	***r* = −0.352 *p* = 0.007**	***r* = −0.284 *p* = 0.031**
PANSS excitement factor	***r* = 0.268 *p* = 0.044**	*r* = 0.120 *p* = 0.373
PANSS depression factor	*r* = 0.101 *p* = 0.450	***r* = −0.289 *p* = 0.028**
PANSS cognitive factor	*r* = −0.041 *p* = 0.762	*r* = −0.220 *p* = 0.097
*PANSS traditional summary scores*		
PANSS positive total	*r* = 0.044 *p* = 0.746	*r* = −0.001 *p* = 0.993
PANSS negative total	***r* = −0.300 *p* = 0.022**	***r* = −0.309 *p* = 0.018**
PANSS general total	*r* = −0.028 *p* = 0.836	***r* = −0.295 *p* = 0.025**

*r*, Pearson correlation coefficient. *N*’s = 57–58. Bolded values are statistically significant correlations at *P* < 0.05.

For further elucidation of some of the correlation structure in relation to effects on odor identification and odor discrimination, we present simplified heatmaps for examination in the (see Supplementry Figures S1–S4).

### Prediction of CSZ versus control group membership

We examined which variables significantly predicted membership in the schizophrenia and control groups. A logistic regression equation with stepwise procedures was used for determining which variables significantly predicted CSZ versus control group membership. In the main analysis we included variables for which we had data values for all subjects, or with only occasional missing values on one or few subjects (odor variables, matric variables, age, sex, smoking). Statistically significant prediction variables in the final model were MATRICS domain scores of speeds of processing and social cognition and odor discrimination score (Table 8), but not odor identification, sex, age, cigarette smoking or other MATRICS variables. The model correctly classified 84.31% of subjects into CSZ versus control groups with sensitivity of 0.8545 and specificity of 0.8298 (See supplement for details of model). When we examined only odor discrimination alone in addition to the background variables (age, sex, cigarettes smoked) in the regression equation, odor discrimination was a significant predictor of CSZ versus control group membership (*β* = 0.417, Wald Ch-Sq 13.484, *p* = 0.0002) with a sensitivity of 0.776 and specificity of 0.750.

**Table 8:** Significant variables discriminating subjects with Schizophrenia from controls.

Analysis of maximum likelihood estimates						Odds ratio estimates		
Parameter	DF	Estimate	Standard error	Wald chi-square	Pr > ChiSq	Point estimate	95% wald confidence limits	
Intercept	1	−12.2012	2.4987	23.8446	<0.0001			
MATRICS speed of processing	1	0.1079	0.0275	15.4479	<0.0001	1.114	1.056	1.176
MATRICS social cognition	1	0.1052	0.0328	10.3023	0.0013	1.111	1.042	1.185
Odor discrimination score	1	0.3472	0.1267	7.5036	0.0062	1.415	1.104	1.814

In additional analysis we examined whether the DNMT or GABAergic mRNAs in lymphocytes significantly predicted group membership. As presented in our previous publications (15) each of the mRNAs was measured in a subset of CSZ and control subjects and the subsets were not completely overlapping for all the markers. Therefore, each mRNA was added in a separate logistic regression comprised of subjects who had values for that mRNA marker as well as the variables in the main logistic regression analysis as described in the preceding paragraph. In these analyses none of the mRNA markers were statistically significant predictor variables in the final models (Wald Chi-Square P’s all >0.05), and the significant differentiating variables remained the same as in the original model above (odor discrimination, speed of processing, social cognition) (see supplement for analysis methods details). In a truncated model with only odor variables and mRNA variables entered into the model (MATRICS variables excluded), odor discrimination was always a significant predictor of group membership, but none of the other mRNAs were a significant predictor in the model, except that in one regression model DNMT3A was also a significant predictor of CSZ versus control group membership in addition to odor discrimination.

## Mediation analysis

To further clarify the influence of some of the variables on odor identification or discrimination, we performed several mediation analyses using the R package discussed in the methods section (for details on the statistical methods see Supplementary data).

In one analysis we assessed whether odor discrimination scores mediated the diagnostic effect (i.e., CSZ versus controls) on odor identification. There is a significant indirect effect of the diagnosis group, on odor identification mediated by odor discrimination (i.e., *β* = −0.632, *p* = 0.002), indicating that odor discrimination mediates the effect on odor identification.

In another analysis we assessed whether any of the mRNA variables mediated the diagnostic group effects on odor identification or odor discrimination. The results showed that none of the selected mRNAs are mediators of the diagnostic group effect of odor identification or odor discrimination (all P’s > 0.05) (see Supplementary Table S3).

In a third analysis we assessed whether the two MATRICS variables, speed of processing and social cognition, which were significant predictors of diagnostic group classification, also mediated the relationship between diagnostic groups’ two odor variables. MATRICS speed of processing fully mediated the relationship between diagnostic group and odor identification (i.e., *β* = −1.001, *p* < 2e-16), and partially mediates the effects between diagnostic group and odor discrimination (i.e., ACME *β* = −0.941, *p* = 0.006 ADE β = −1.442, *p* = 0.022). MATRICS social cognition did not mediate the diagnostic group effect on odor identification (i.e., β = 0.107, *p* = 0.66) or odor discrimination (i.e., *β* = −0.1201, *p* = 0.67).

**Figure 3:**
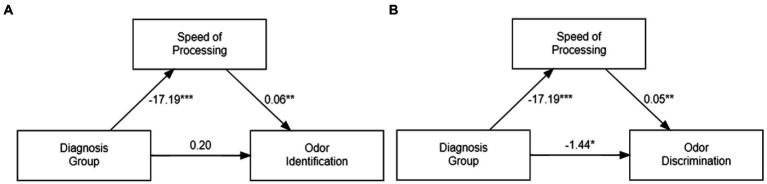
Graphical Representation of Mediation Effects of MATRICS Speed of Processing Score on Odor Identification **(A)** and Odor Discrimination **(B)**. Each number is the β value from the mediation analysis of either the *direct effect* (ADE) of diagnostic group (schizophrenia vs. control) on odor variables controlling for the mediator and covariates (age, sex and number of cigarettes smoked) (*bottom arrow*), or the *indirect mediation effect* (ACME) (*the product of the two side arrows*), Statistical significance of β value: * *p* < 0.05,** *p* < 0.01, ***p < 0.001.

Figure 3 presents graphical representations of the mediation effects of speed of processing on odor identification and odor discrimination.

## Discussion

The current study showed that odor discrimination scores were significantly lower in CSZ than controls (*p* < 0.001) and that odor discrimination significantly (*p* = 0.0002) predicted a subject’s group member in chronic schizophrenia versus control group with a Sensitivity of 0.78 and Specificity of 0.75. In CSZ subjects, scores on odor discrimination were negatively correlated with GABAergic mRNA levels in peripheral lymphocytes.

DNA methyltransferases (DNMTs) regulate many aspects of memory formation. DNMTs have been associated with processing of olfactory stimuli in animal studies and shown to be involved in forming specific odor memories in honeybees (7). Blocking DNA methyltransferases affected odor specificity of the memory (memory discriminatory power) (26). Mice deficient in GAD67 (enzyme that catalyzes the decarboxylation of glutamate to GABA) have been shown to be less sensitive to social and non-social odors (8) and reeler mice deficient in GAD67 required more training sessions to learn correct odor discrimination (9). However, the specific neuronal pathways mediating these influences of DNMTs and GABA are not fully elucidated.

This is the first study to assess the relationship of DNMT and GABAergic mRNAs to odor identification and discrimination in human lymphocytes. Previous studies from our research group have described increased DNMT1 mRNA and decreased GAD67 mRNA in the brains of patients with schizophrenia, and our research also shows increased DNMT1 mRNA in the patients’ lymphocytes (12–17). Our current data showed a negative correlation between DNMT1 mRNA and odor identification (Table 4) and a negative correlation between GABAergic mRNAs (GAD1, GAD25, GAD67) and odor discrimination (Table 5). For odor discrimination, the GABAergic negative correlations were significant for CSZ subjects but not for controls. From animal studies, it would have been expected that higher GAD levels would facilitate odor discrimination, whereas our data shows that CSZ with lower GABAergic mRNAs had better odor discrimination. It is possible that lymphocyte GAD mRNAs are not representative of brain levels. We have previously reported (15) that, whereas GAD_67_ mRNA was significantly lower in the frontal cortex of post-mortem brain samples of CSZ versus control, GAD1 mRNA was higher in lymphocytes of CSZ compared to control and GAD_67_ mRNA showed no difference between the two groups. If we could measure levels of GABAergic and DNMT mRNAs in brain or peripheral olfactory neurons in conjunction with odor identification or discrimination in CSZ and control subjects, we could examine more definitely whether higher levels of these mRNAs are involved in facilitating odor identification and discrimination in humans. The lack of significant mediation effects of the mRNA variables on the CSZ versus control group effects on odor identification or odor discrimination is consistent with their lack of effects as predictor variables for group membership and raises questions about the substantive interpretation of the correlations with these peripheral markers.

Researchers in the University of Pennsylvania group have presented extensive research on structural and electrophysiological abnormalities in the olfactory system of patients with schizophrenia- those at high risk of developing schizophrenia and their first-degree relatives (1, 5). They present behavioral evidence implicating a disruption of cyclic AMP mediating signal transduction in the olfactory system in patients with schizophrenia and those at risk for this disorder (4, 27).

Fewer studies have investigated odor discrimination in schizophrenia (28–31) and compared the deficits in odor discrimination to odor identification. In the current study odor discrimination showed the strongest difference and largest effect size in comparing CSZ to controls and odor identification was not significantly different between CSZ and controls. Odor discrimination, along with two MATRICS variables, but not odor identification, significantly predicted group membership in CSZ versus controls. Rupp and associates also reported that odor discrimination had the strongest significance for difference (*p* < 0.001) between patients with schizophrenia and controls, although they did not compute effect size (31). Ugur and associates found that deficits in odor discrimination, but not odor identification differentiated monozygotic twins with schizophrenia from healthy twins (32). Significant deficits in odor discrimination have been recently reported in first-episode schizophrenia compared to controls (33) and scores on this measure were significantly related to social cognition in face processing (34). Similar to the results of the current study, odor discrimination was one of three variables significantly predicting schizophrenia versus control group membership in a first episode study (35). The specificity of odor discrimination’s relation to brain structures in schizophrenia is highlighted in a recently published preliminary study (34). In this study by Etyemez and colleagues, they found odor discrimination in first-episode patients with schizophrenia significantly correlated with the volume of a structure in the frontal cortex (superior frontal gyrus) and connections between this structure and other specific brain regions in the occipital lobe.

Sex differences may be important in differentiating selective olfactory function deficits in chronic schizophrenia versus controls. Although there was no overall difference in odor identification between CSZ and controls in our total sample, male CSZ showed lower scores on odor identification than controls (*p* = 0.032), whereas for females there was no difference. For odor discrimination however, both males and females showed a similar difference with CSZ lower than controls. Malaspina reported that males had lower odor identification scores than females on the USPIT, but there was no significant diagnosis x sex interaction effect (36, 37). Kamath and associates, however, found no significant effects of sex or interaction of sex x diagnosis effects in first episode psychosis patients for odor identification or odor discrimination measures (33).

Although several studies have investigated the relationship of odor deficits to cognitive deficits, to our knowledge this is the first study to utilize the MATRICS battery to investigate this question. Although there was a correlation of several MATRICS domain scores and odor deficits, further analysis showed statistically significant correlation in the CSZ sample but not the controls. Furthermore, consistent with the findings from Malaspina’s previous study (37) these correlations appeared to be higher in female CSZ. There was a significant sex effect for the correlations with some MATRICS domain scores and odor discrimination scores. The relative importance of MATRICS speed of processing and social cognition scores are highlighted because only these two domain scores significantly predicted group CSZ versus control group membership. Mediation analysis also showed that speed of processing significantly mediated the relationship between diagnostic group effects and odor identification or discrimination.

This is the first study to correlate odor scores in schizophrenia with PANSS derived factors from the 5-factor PANSS model, and this provides additional information than the standard PANSS scale summary scores. The significant correlation of PANSS Negative symptom factor with poorer odor identification and discrimination in schizophrenia is consistent with previous studies linking odor deficits in schizophrenia to PANNS negative symptoms or the deficit syndrome type of schizophrenia (29, 33, 38–40). However, one meta-analysis indicated that the effect size of the difference in odor scores between controls and schizophrenia was not meditated by the extent of negative symptoms (1). The unexpected positive correlation between PANSS Excitement factor and odor identification scores warrants further exploration.

## Limitations

The small number of females, especially in our CSZ sample, makes our results of the effects of gender on odor scores less robust. Furthermore, there were differences in the characteristics of the 8 CSZ females in our sample compared to the 16 controls. The mean age of the CSZ females was 45.4 and most were in their menopausal or post-menopausal period, whereas the age of the control females was younger, mean age 34.7, and only 3 could be considered in their postmenopausal stage. Previous research has shown there are differences in incidence and symptoms in female patients with schizophrenia, which may be related to their pre- versus post-menopausal state mediated by changes in estrogen and related hormones. It is possible that a CSZ sample with a larger number of younger females could have shown more pronounced sex differences on some of our measures. The strongest correlation of a DNMT1 variant with odor identification was based on a small sample and may make this result less reliable. The mRNA levels in lymphocytes may not reflect brain levels, or levels of neuronal tissue which may extend into the nasal epithelium, and therefore our negative correlations between odor identification or discrimination with DNMT or GABAergic makers may not reflect their true relationship in neural tissue. We did not measure odor sensitivity threshold or some other measures of odor perception, so we cannot conclude that odor discrimination is the most powerful odor measure differentiating patients with schizophrenia from controls. The lack of information on education levels in our subjects may make the interpretation of MATRICS score differences less clear. The lack of precise data on length of illness in the CSZ subjects is another limitation.

## Conclusion

Odor discrimination was a more powerful variable than odor identification in discriminating CSZ from controls and should be used more regularly as an odor measure in studies of schizophrenia. Odor discrimination scores along with two MATRICS domain scores (speed of processing and social cognition) were significant variables in logistic regression analysis and when combined they correctly classified 84% of the subjects into CSZ or control groups. Male versus female gender was an important factor in differentiating odor identification scores in CSZ versus controls, but this factor was not an important differentiator for odor discrimination. The substantive meaning of the negative correlations of odor discrimination and GABAergic mRNA variables in peripheral lymphocytes of CSZ needs more investigation and comparison with results in neural tissue.

## Data availability statement

The raw data supporting the conclusions of this article will be made available by the authors, without undue reservation.

## Ethics statement

The studies involving human participants were reviewed and approved by Nathan Kline Institute for Psychiatric Research IRB. The patients/participants provided their written informed consent to participate in this study.

## Author contributions

RS, HS, JD, and AG primarily designed the study, as part of a grant proposal which AL and HJ also participated in the design. HS performed the assays. MY helped carry out the smell tests and other clinical aspects of the study and initial data organization. RS, JD, MZ, and AC statistically analyzed the data. RS, HS, and JD wrote the first draft of the paper. All the authors reviewed the paper, made additions or corrections, and approved the final version.

## Funding

This research was supported in part by NIH grant 1R01MH101043, AG PI, and a philanthropic grant to RS’s section at NKI.

## Conflict of interest

The authors declare that the research was conducted in the absence of any commercial or financial relationships that could be construed as a potential conflict of interest.

## Publisher’s note

All claims expressed in this article are solely those of the authors and do not necessarily represent those of their affiliated organizations, or those of the publisher, the editors and the reviewers. Any product that may be evaluated in this article, or claim that may be made by its manufacturer, is not guaranteed or endorsed by the publisher.

## Supplementary material

The Supplementary material for this article can be found online at: https://www.frontiersin.org/articles/10.3389/fpsyt.2023.1115399/full#supplementary-material
