# Supplementary material for: Deficits in odor discrimination versus odor identification in patients with schizophrenia and negative correlations with GABAergic and DNA methyltransferase mRNAs in lymphocytes
Source: Front Psychiatry. 2023 Mar 28;14:1115399. doi: 10.3389/fpsyt.2023.1115399 (PMC10088370; doi:10.3389/fpsyt.2023.1115399)

**Supplementary Data**

1. **Additional information regarding subject selection**

If a clinical chart diagnosis at one time indicated schizoaffective disorder rather than schizophrenia, but the subject fulfilled other study admission criteria, the patient’s symptoms and history were reviewed carefully by the senior research psychiatrist (RS) to confirm the criteria for schizophrenia diagnosis by strict DSM criteria.

Control subjects were screened on the phone and interviewed in person for current symptoms, mental health and medical history, current medications, and drug history. Controls recruited from the NKI outpatient research subject pool were also reviewed for prior SCID interview diagnostic record when available.

Subjects were excluded if they were heavy cigarette smokers, smoking 1 pack or more of cigarettes/day, or used a daily nicotine patch or nicotine gum because earlier research showed that nicotine or smoking could reduce DNMT mRNA levels.

1. **Comparison of background characteristics of subjects in full sample compared to the subsets of subjects who had values for DNMT1 or GAD1 mRNA.**

Tables S1 and S2 on the following pages below show that there were no significant differences in subject characterizes in the full sample compared to reduced samples, except for one comparison showing that there were slightly fewer subjects on clozapine in the DNMT1 mRNA sample.

| **Characteristic** | **Treatment Group** | **Reduced Sample**  n = 58  (Schizophrenia n=28  Control n=30) | **Total Sample**  n = 106  (Schizophrenia n=58  Control n=48) | **Test** |
| --- | --- | --- | --- | --- |
| Age (m) | Schizophrenia | 45.46$\pm$8.89 | 44.72$\pm$9.62 | T=0.56, df=56, P=0.576 |
|  | Control | 33.63$\pm$10.08 | 35.60$\pm$10.90 | T=1.65, df=46, P=0.106 |
|  |  | T=4.73, df=56, P<0.001 | T=4.57, df=104, P<0.001 |  |
| Sex (M/F) (n) | Schizophrenia | 22/6 | 50/8 | $\chi^{2}$ = 2.65, df = 1, P=0.103 |
|  | Control | 18/12 | 32/16 | $\chi^{2}$ = 1.60, df = 1, P=0.206 |
|  |  | $\chi^{2}$ = 2.33, df = 1, P= 0.127 | $\chi^{2}$ = 5.73, df = 1, P= 0.017 |  |
| Cigarette smoked/week (m) | Schizophrenia | 27.64$\pm$43.10 | 36.16$\pm$45.00 | T=1.40, df=56,  P= 0.166 |
|  | Control | 11.67$\pm$24.96 | 11.81$\pm$27.78 | T=0.05, df=46, P=0.963 |
|  |  | T=1.74, df=56, P=0.087 | T=3.27, df=104, P=0.002 |  |
| On Clozapine (Y/N) (n) | Schizophrenia | 9/19 | 27/31 | $\chi^{2}$ = 4.52, df = 1, P=0.034 |
|  | Control | NR | NR | NR |
|  |  | NR | NR |  |
| PANSS Total (m) | Schizophrenia | 71.93$\pm$18.21 | 71.64$\pm$15.07 | T=0.14, df=56,  P= 0.889 |
|  | Control | NR | NR | NR |
|  |  | NR | NR |  |
| MATRICS overall composite (m) | Schizophrenia | 21.54$\pm$14.42 | 21.45$\pm$12.57 | T=0.05, df=53, P=0.963 |
|  | Control | 40.10$\pm$9.33 | 41.53$\pm$9.76 | T=1.35, df=45, P=0.185 |
|  |  | T=5.79, df=54, P<0.001 | T=8.89, df=100, P<0.001 |  |

**Table S1. Characteristics of subjects of total sample and subjects of reduced sample with DNMT1 measured.**

NR=Not relevant or not available; (n)=number of subjects, m= Mean ± S.D., (Y/N) = Yes/No. (M/F) = male/female. Statistical tests: $\chi^{2}$ =chi-square, T= t-test.

| **Characteristic** | **Treatment Group** | **Reduced Sample**  n = 65  (Schizophrenia n=35  Control n=30) | **Total Sample**  n = 106  (Schizophrenia n=58  Control n=48) | **Test** |
| --- | --- | --- | --- | --- |
| Age (m) | Schizophrenia | 45.51$\pm$9.37 | 44.72$\pm$9.62 | T=0.77, df=56, P=0.445 |
|  | Control | 33.63$\pm$10.08 | 35.60$\pm$10.90 | T=1.65, df=46, P=0.106 |
|  |  | T=4.92, df=63, P<0.001 | T=4.57, df=104, P<0.001 |  |
| Sex (M/F) (n) | Schizophrenia | 28/7 | 50/8 | $\chi^{2}$ = 2.86, df = 1, P= 0.091 |
|  | Control | 18/12 | 32/16 | $\chi^{2}$ =1.6, df = 1,  P= 0.206 |
|  |  | $\chi^{2}$ = 3.12, df = 1, P= 0.077 | $\chi^{2}$ = 5.73, df = 1, P= 0.017 |  |
| Cigarette smoked/week (m) | Schizophrenia | 30.51$\pm$44.23 | 36.16$\pm$45.00 | T=1.18, df=56, P=0.242 |
|  | Control | 11.67$\pm$24.96 | 11.81$\pm$27.78 | T=0.05, df=46, P=0.963 |
|  |  | T=2.07, df=63, P=0.043 | T=3.27, df=104, P=0.002 |  |
| On Clozapine (Y/N) (n) | Schizophrenia | 14/21 | 27/31 | $\chi^{2}$ =1.52, df = 1, P= 0.217 |
|  | Control | NR | NR | NR |
|  |  | NR | NR |  |
| PANSS Total (m) | Schizophrenia | 72.31$\pm$17.27 | 71.64$\pm$15.07 | T=0.42, df=56, P=0.677 |
|  | Control | NR | NR | NR |
|  |  | NR | NR |  |
| MATRICS overall composite (m) | Schizophrenia | 21.52$\pm$14.09 | 21.45$\pm$12.57 | T=0.04, df=53, P=0.966 |
|  | Control | 40.10$\pm$9.33 | 41.53$\pm$9.76 | T=1.35, df=45, P=0.185 |
|  |  | T=6.11, df=61, P<0.001 | T=8.89, df=100, P<0.001 |  |

**Table S2.** **Characteristics of subjects of total sample and subjects of reduced sample with GAD1 measured**

NR=Not relevant or not available; (n)=number of subjects, m= Mean ± S.D., (Y/N) = Yes/No. (M/F) = male/female. Statistical tests: $\chi^{2}$ =chi-square, T= t-test.

**Details of Statistical Methods**

**3. Multiple Regression Analysis of reduced mRNA sample characteristic effects on the correlations between age, sex, number of cigarettes smoked, diagnosis, and odor identification or discrimination**

This analysis aims to examine the effects of reduced mRNA sample characteristics (i.e., whether the subject has the selected mRNA variable measured) on the correlations between age, sex, the number of cigarette smoke, diagnosis, and odor identification or discrimination. In this analysis, we have selected two mRNA variables, DNMT1 and GAD1. Since only selected subjects to have certain mRNA variables measured in our data, if the subject has the selected mRNA variable measured, he/she is considered as in the reduced sample. Four multiple regression models were examined, with predicting variables age, sex, number of cigarettes smoked, diagnosis group (i.e., schizophrenia and control group), reduced sample characteristics (i.e., whether the subject is present in the reduced sample), and the interaction term of diagnosis and reduced sample characteristics, and outcome variables as either odor discrimination or odor identification. The statistical significance level for this analysis was set to 0.05. In this analysis, we mainly focus on the interaction terms (i.e., whether the interaction terms are statistically significant in predicting odor variables). If the p-value of the interaction term is less than or equal to 0.05, then we reject the null hypothesis and conclude that there is a statistically significant relationship between the interaction term and the response variable. On the other hand, if the p-value is greater than 0.05, we fail to reject the null hypothesis and conclude that the relationship between the interaction term and response variable is not statistically significant.

**4. Multiple Regression Analysis of sex effects on the correlations between mRNA variables and odor identification or discrimination**

This analysis aims to examine the effects of sex on the correlations between selected mRNA variables (i.e., GABAergic and DNMT1 variables) and odor identification or odor discrimination. In this analysis, the data was limited to only include schizophrenia patients. Two multiple linear regression models were built with each of the selected mRNA variables, sex, and their interaction term, one with odor identification as the response variable and the other with odor discrimination as the response variable. The statistical significance level was set to 0.05. In regression analysis, p-values determine whether there is a statistically significant relationship between each predictor and response variable. In this analysis, we mainly focus on the interaction terms (i.e., whether the interaction terms are statistically significant in predicting odor variables). If the p-value of the interaction term is less than or equal to 0.05, then we reject the null hypothesis and conclude that there is a statistically significant relationship between the interaction term and response variable (i.e., there is a sex effect on the correlations between the selected mRNA variables and odor variables). On the other hand, if the p-value is greater than 0.05, then we fail to reject the null hypothesis and conclude that the relationship between the interaction term and response variable is not statistically significant (i.e., there is no sex effect on the correlations between the selected mRNA variables and odor variables). If a sex effect was observed from the multiple regression analysis, then the Pearson correlation coefficients between the selected mRNA variables and odor variables for both males and females will be investigated to examine the difference in sex effect on the correlations of those variables.

**2.2 Multiple Regression Analysis of sex effects on the correlations between MATRICS variables and odor identification or discrimination**

This analysis aims to examine the effects of sex on the correlations between MATRICS variables (i.e., speed of processing, attention vigilance, working memory, verbal learning, visual learning, social cognition, reasoning-problem solving, and overall composite score) and odor identification or odor discrimination. In this analysis, the data was limited to only include schizophrenia patients. Two multiple linear regression models were built with each MATRICS variable, sex, and their interaction term, one with odor identification as the response variable and the other with odor discrimination as the response variable. The statistical significance level for this analysis was set to 0.05. In this analysis, we mainly focus on the interaction terms (i.e., whether the interaction terms are statistically significant in predicting odor variables). If the p-value of the interaction term is less than or equal to 0.05, then we reject the null hypothesis and conclude that there is a statistically significant relationship between the interaction term and response variable (i.e., there is a sex effect on the correlations between the MATRICS variables and odor variables). On the other hand, if the p-value is greater than 0.05, then we fail to reject the null hypothesis and conclude that the relationship between the interaction term and response variable is not statistically significant (i.e., there is no sex effect on the correlations between the MATRICS variables and odor variables). If a sex effect was observed from the multiple regression analysis, then the Pearson correlation coefficients between the MATRICS variables and odor variables for both males and females will be investigated to examine the difference in sex effect on the correlations of those variables.

**5. Correlation Coefficient Comparison of independent samples**

We have conducted a correlation comparison with two independent samples in this paper. Since the correlations were computed on the same variables in two independent samples (e.g., correlation of mRNA variables between schizophrenia and control group, correlation of mRNA variables between male and female), we wanted to test the hypothesis of whether each of the two population correlations are the same (i.e., $H_{0}:\rho_{1}=\rho_{2}$ vs. $H_{1}:\rho_{l}\neq\rho_{2}$). To test this hypothesis, we first applied the Fisher’s (1921) r-to-z transformation to the two Pearson correlation coefficients, $r_{1}$ and $r_{2}$, to convert the two sample correlations into r’ values and S_r’,_ the standard error of r′.


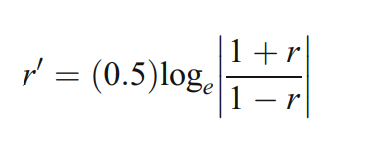


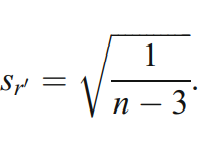


Where n is the sample size.

Then we applied the Z-test to test the difference between the two sample correlations. Z score was calculated using the following formula:


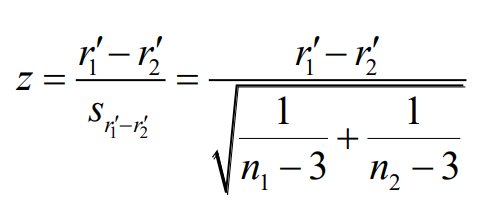


Where ${r'}_{1}$ and ${r'}_{2}$ are the r’ values of the Pearson correlation coefficients, $r_{1}$ and $r_{2}$, and $n_{1}$ and $n_{2}$ are the sample sizes of $r_{1}$ and $r_{2}$.

Finally, we obtained the p-values from the Z score. The statistical significance level for this analysis was set to 0.05. If the p-value is less than or equal to 0.05, we reject the null hypothesis and conclude that there is a significant difference between the two sample correlations. On the other hand, if the p-value is greater than 0.05, we fail to reject the null hypothesis and conclude that there is not enough evidence to show a significant difference between the two sample correlations.

**6. Main Analysis: Logistic Regression Analysis for selecting variables which significantly predict group membership in schizophrenia and control group**

The main objective of this analysis is to identify variables that have significant effects in predicting schizophrenia (CSZ) or control membership and to determine the relative strength of each variable. Two logistics regression models were examined in this analysis, with predicting variables odor identification, odor discrimination, MATRICS domain variables, age, sex, and two different smoking variables, smoker/non-smoker status and the number of cigarettes smoked per week, respectively (i.e., Model 1: Logistics regression with variable Smoker/Non-smoker Status and Model 2: Logistics regression with Variable Number of Cigarettes Smoked Per Week). Missing values were handled using listwise deletion. The final model was selected using stepwise selection and model comparison. Multicollinearity investigation was performed using Pearson Correlation Coefficient, Variance Inflation Factor (VIF), and Tolerance. Global Null Hypothesis Test and Hosmer and Lemeshow Goodness-of-Fit Test were used to test the model fitting. The final model performance was reported using the accuracy rate and the area under the curve (AUC, or the C-statistic) from ROC Curve. Three methods (i.e., standard coefficients, c-statistics, and logistic pseudo partial correlation), described in Doug Thompson’s paper, were used to determine the variable importance ranking of the predictors from the final selected model. The final variable importance ranking was determined by comparing the results of the three methods.

**6.1 Result and model interpretation of main analysis for variables which predict classification into schizophrenia or control diagnostic groups**

Model Interpretation

This analysis examined two logistics regression models (i.e., Model 1 and Model 2). The stepwise selection was used to determine the most important variables in predicting schizophrenic or control membership. Based on the results of both Model 1 and Model 2, only odor discrimination, MATRICS cognitive measures social cognition, and MATRICS cognitive measures speed of processing are significant variables (i.e., all p-values < 0.05).


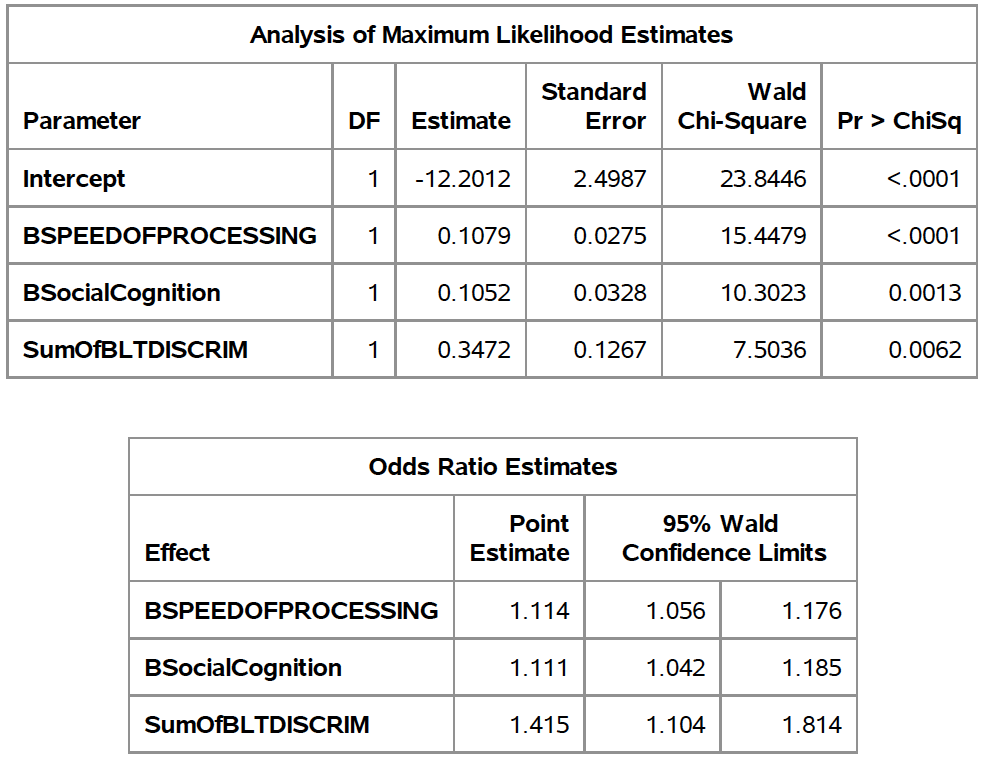


As shown in the tables above, all predictors are positively associated with the classification of schizophrenia patients and controls. For one unit increase in MATRICS cognitive measures speed of processing, the odds of being in the schizophrenia group increases by 1.114 times, adjusted for all other predictors. For one unit increase in MATRICS cognitive measures social cognition, the odds of being in the schizophrenia group increase by 1.111 times, adjusted for all other predictors. For one unit increase in odor discrimination, the odds of being in the schizophrenia group increase by 1.415 times, adjusted for all other predictors.

Model Performance

The final model performance was reported using the accuracy rate and the area under the curve (AUC, or the C-statistic) from ROC Curve. Based on the confusion matrix below, this model has correctly classified 47 of 55 schizophrenia patients and 39 of 47 controls from the dataset, resulting an accuracy rate of 0.8431. This indicates that 84.31% of the participants are correctly classified by this model. It also has a sensitivity rate of 0.8545 and a specificity of 0.8298. This indicates that 85.45% of the participants are correctly identified as schizophrenia patients, and 82.98% of the participants are correctly identified as controls. This final model has an AUC of 0.9226, indicating a 92.26% chance that the selected model will be able to distinguish between schizophrenia and controls.


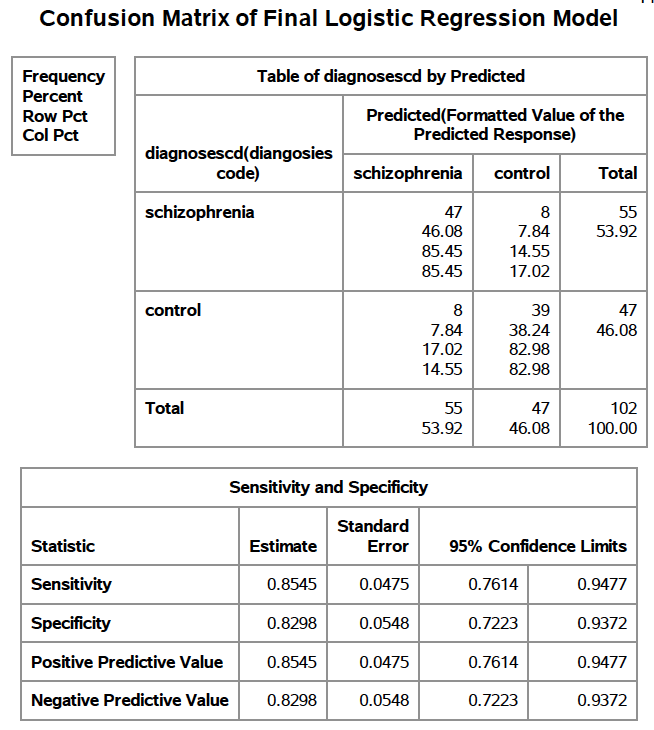

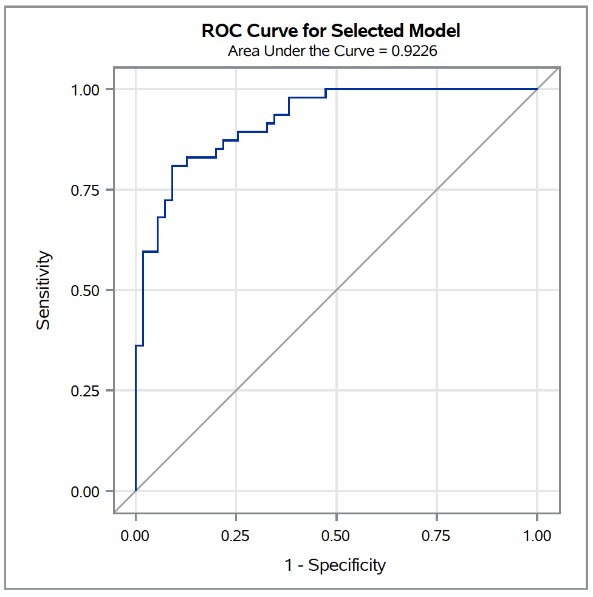


Variable Importance Ranking

There is no one best method for measuring the variable importance of predictors in the logistic regression model. In this analysis, three methods (i.e., standardized regression coefficients, concordance statistic (c-statistics), and logistic pseudo partial correlation), described in Doug Thompson’s paper, were used and compared to determine the final variable importance ranking. The first method used is the standardized regression coefficients method. It can be used to measure the strength of association of the predictors with the outcome. The higher the standardized coefficients indicate, the higher the strength of association with schizophrenic and control classification. The second method used is the logistic pseudo partial correlation method. This method measures the marginal association between predictors and outcomes (i.e., the closer the logistic pseudo partial correlation to 1 or -1, the stronger the marginal association), considering the other predictors. The last method used in this analysis is the c-statistics method. C-statistics is used to determine the model’s ability to classify outcomes correctly. Therefore, the predictors can be ranked by computing a separate model for each predictor, estimating and expressing the c-statistics for each model in terms of the absolute value of their differences from 0.5. This difference measures the efficacy of a predictor in classifying schizophrenia and control membership.


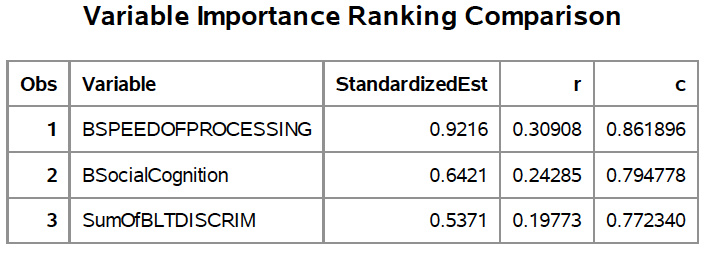


Based on standard regression coefficients, c-statistics, and logistic pseudo partial correlation, the most important variables in predicting schizophrenic (CSZ) or control membership is MATRICS cognitive measures of speed of processing, followed by MATRICS cognitive measures of social cognition, and odor discrimination.

**7. Additional Analysis: Logistic Regression Analysis for prediction of schizophrenia and controls with selected mRNA variable**

The main objective of this analysis is to examine whether the selected mRNA variables have significant effects in predicting schizophrenia (CSZ) or control membership and determine each variable's relative strength. Two logistics regression models were examined for each mRNA variable in this analysis, one with predicting variables odor identification, odor discrimination, and selected mRNA variable (i.e., Model 1), and the second one with predicting variables odor identification, odor discrimination, MATRICS domain variables, age, sex, the number of cigarettes smoked per week and selected mRNA variable (i.e., Model 2). Missing values were handled using listwise deletion. For each model, multicollinearity investigation and model fitting test were performed using the same methods as in the main analysis. The stepwise selection was performed on each model to determine the statistically significant variables in classifying schizophrenia and controls. Finally, variable importance ranking comparison was performed on each model, using the same methods as in the main analysis (i.e., standard coefficients, c-statistics, and logistic pseudo partial correlation).

**8. Causal Mediation Analysis with odor discrimination**

In this paper, mediation analyses were performed with an R package named Mediation, using the model-based approach. This analysis examines whether odor discrimination mediates the effect of diagnosis on odor identification. Two models were fitted in this analysis, the mediator model, where the odor discrimination score is modeled with predicting variables diagnosis group and covariates (i.e., age, sex, and the number of cigarettes smoked), and the outcome model, where odor identification score is modeled with predicting variables diagnosis group, the potential mediator odor discrimination and covariates (i.e., age, sex, and the number of cigarettes smoked). Both models were fitted with ordinary least squares regression. Missing values were handled using listwise deletion. The mediate function was used to estimate the average causal mediation effect (ACME) and the average direct effect (ADE) of the fitted models, with the default number of simulations (i.e., sims = 1000) and the default simulation type (i.e., quasi-Bayesian Monte Carlo method based on normal approximation). The statistical significance level for this analysis was set to 0.05. If the p-value of ACME is less than or equal to 0.05, then we conclude that there is a significant mediation effect of odor discrimination between odor identification score and diagnosis, controlling for covariates. On the other hand, if the p-value of ACME is greater than 0.05, then we conclude that there is not enough evidence to show a significant mediation effect of odor discrimination between odor identification score and diagnosis, controlling for covariates. If the p-values of both ACME and ADE are significant, we conclude that there is a partial mediation of odor discrimination between odor identification and diagnostic effect. Alternatively, if only ACME is significant but not ADE, we conclude that there is a full mediation.

**9. Causal Mediation Analysis with MATRICS variables or mRNA variables**

In this paper, mediation analyses were performed with an R package named Mediation, using the model-based approach. This analysis aims to examine whether the selected MATRICS cognitive measures or the selected mRNA variables mediate the effect of diagnosis on odor identification or odor discrimination. Two models were fitted in this analysis, the mediator model, where MATRICS cognitive measures or the selected mRNA variables are modeled as the function of diagnosis group and covariates (i.e., age, sex, and the number of cigarettes smoked), and the outcome model, where odor identification score or odor discrimination score, is modeled as the function of diagnosis group, the potential mediator(i.e., the MATRICS cognitive measures, or the selected mRNA variables) and covariates (i.e., age, sex, and the number of cigarettes smoked). Both models were fitted with ordinary least squares regression. Missing values were handled using listwise deletion. The mediate function was used to estimate the average causal mediation effect (ACME) and the average direct effect (ADE) of the fitted models, with the default number of simulations (i.e., sims = 1000) and the default simulation type (i.e., quasi-Bayesian Monte Carlo method based on normal approximation). The statistical significance level for this analysis was set to 0.05. If the p-value of ACME is less than or equal to 0.05, then we conclude that there is a significant mediation effect of the selected MATRICS cognitive measures or the selected mRNA variables between odor identification score or odor discrimination score and diagnosis, controlling for covariates. On the other hand, if the p-value of ACME is greater than 0.05, then we conclude that there is not enough evidence to show a significant mediation effect of the selected MATRICS cognitive measures or the selected mRNA variables between odor identification score or odor discrimination score, and diagnosis, controlling for covariates. If the p-values of both ACME and ADE are significant, we conclude that there is a partial mediation of the selected variables between odor identification and diagnostic effect. Alternatively, if only ACME is significant but not ADE, we conclude that there is a full mediation.

**S3. Lack of significant mediation by mRNA variables - Average causal mediation effects (ACME) of Mediation Analysis with Diagnosis Group (Exposure), mRNA Variables (Mediator) and Odor Variables (Outcome)**

|  | **Odor Identification** | | | | **Odor Discrimination** | | | |
| --- | --- | --- | --- | --- | --- | --- | --- | --- |
| **mRNA Variables** | **Estimate** | **95% CI** | | **P-value** | **Estimate** | **95% CI** | | **P-value** |
|  |  | LL | UL |  |  | LL | UL |  |
| **DNMT1 (log transformed)**  **(n = 58)** | -0.055 | -0.53 | 0.37 | 0.79 | -0.012 | -0.29 | 0.24 | 0.93 |
| **DNMT3A**  **(n = 65)** | 0.043 | -0.42 | 0.51 | 0.87 | 0.237 | -0.19 | 0.82 | 0.28 |
| **GAD1 (log transformed)**  **(n = 65)** | -0.001 | -0.32 | 0.34 | 0.99 | -0.375 | -1.03 | 0.07 | 0.14 |
| **GAD25 (log transformed)**  **(n = 68)** | -0.083 | -0.44 | 0.17 | 0.52 | -0.203 | -0.80 | 0.27 | 0.38 |
| **GAD67**  **(n = 68)** | -0.011 | -0.22 | 0.17 | 0.90 | 0.007 | -0.18 | 0.20 | 0.92 |

All models were controlled for covariates age, sex and number of cigarettes smoked.

**References**

Bruce Weaver, Karl L. Wuensch. SPSS and SAS programs for comparing Pearson correlations and OLS regression coefficients. *Behav Res* (2013) 45:880–895.

Thompson, D., Wi, M. and Health, A. (2009) LR, Ranking Predictors in Logistic
Regression. Paper D10-2009, Assurant Health, West Michigan, 1-13.
*- MWSUG*. Retrieved Feb 4, 2023, from: <https://www.mwsug.org/proceedings/2009/stats/MWSUG-2009-D10.pdf>

Tingley, D., Yamamoto, T., Hirose, K., Keele, L., & Imai, K. (2014). mediation: R Package for Causal Mediation Analysis. Journal of Statistical Software, 59(5), 1–38. https://doi.org/10.18637/jss.v059.i05

**10**. **Simplified Heatmaps of Correlations**


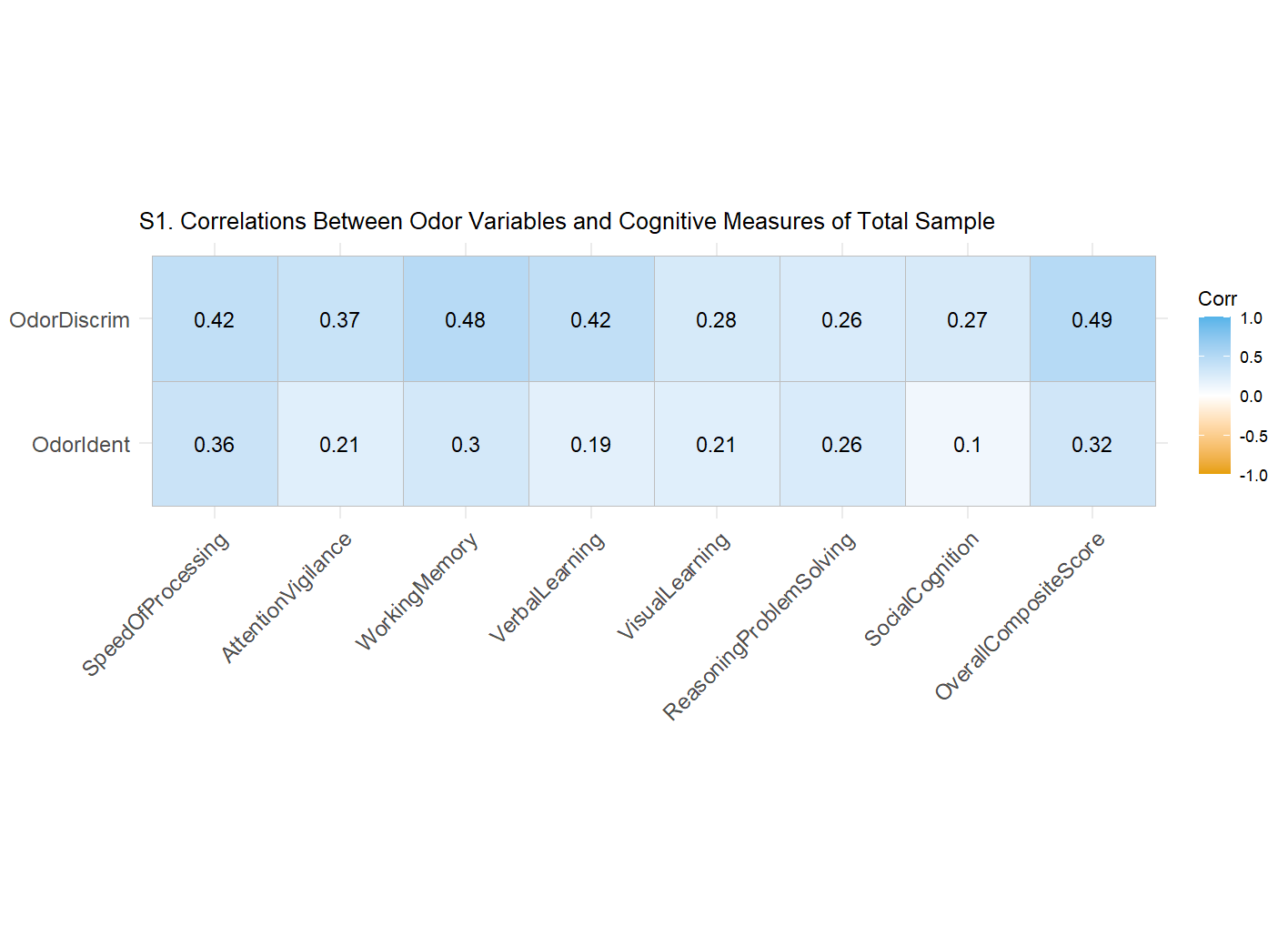


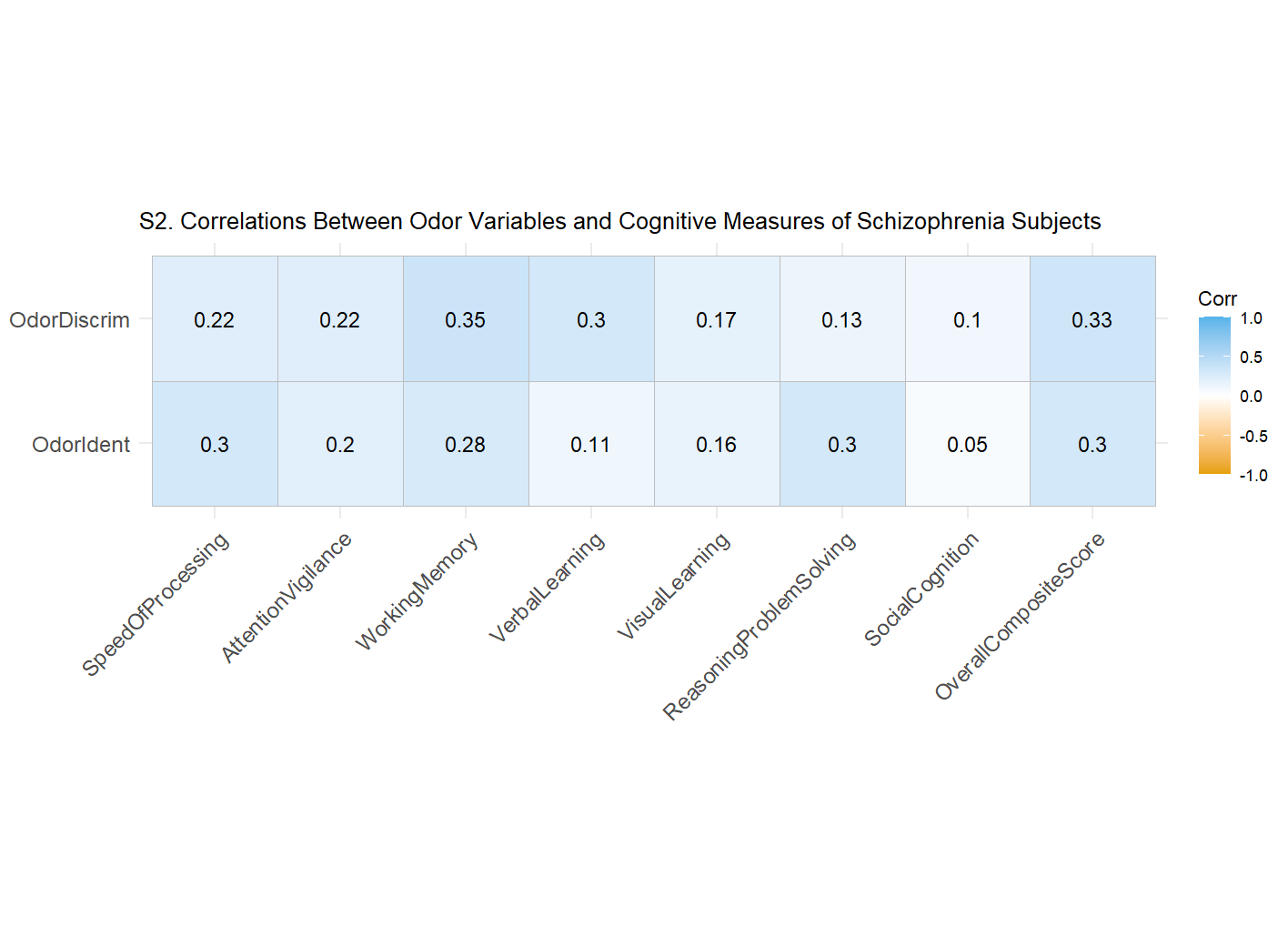


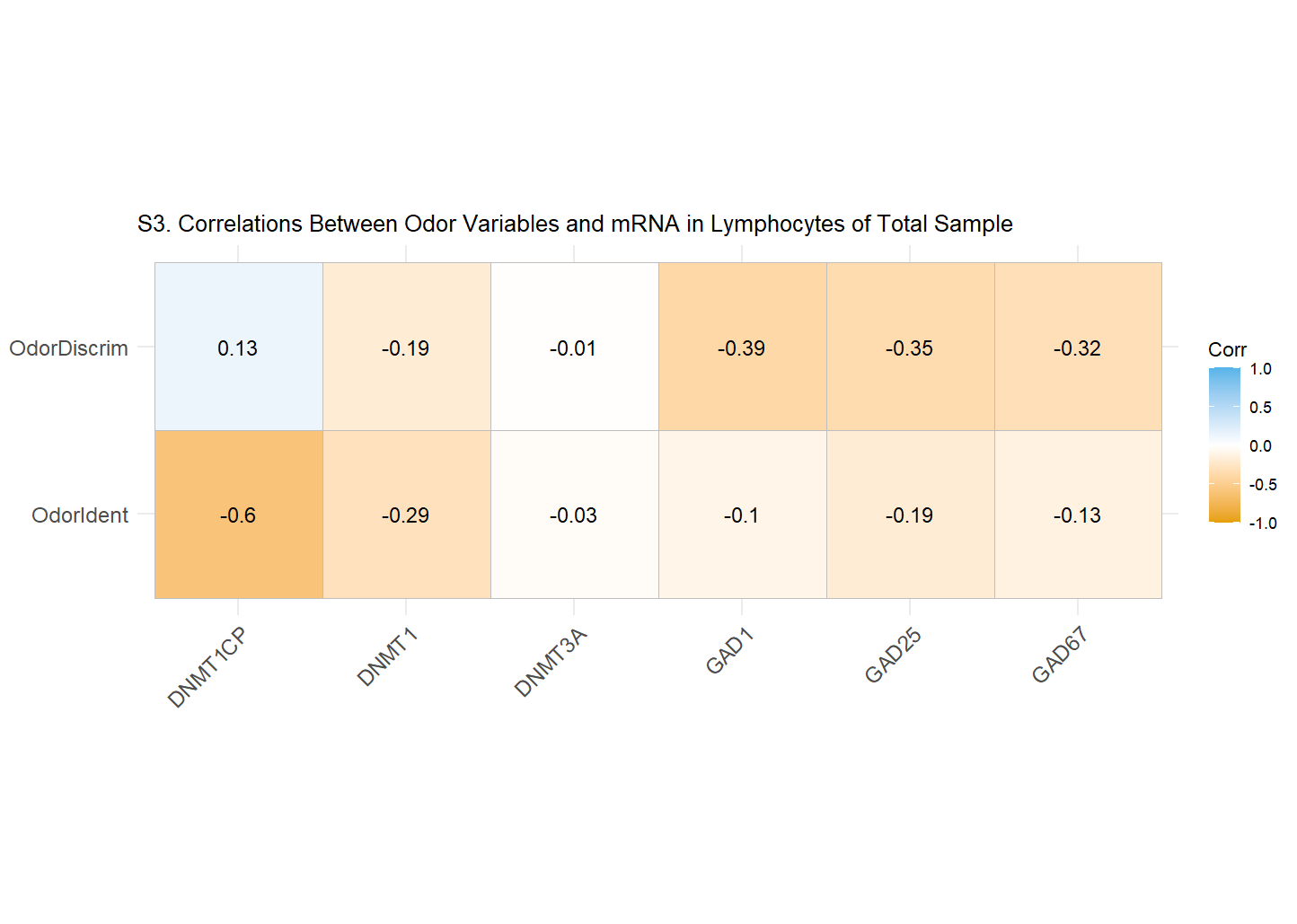


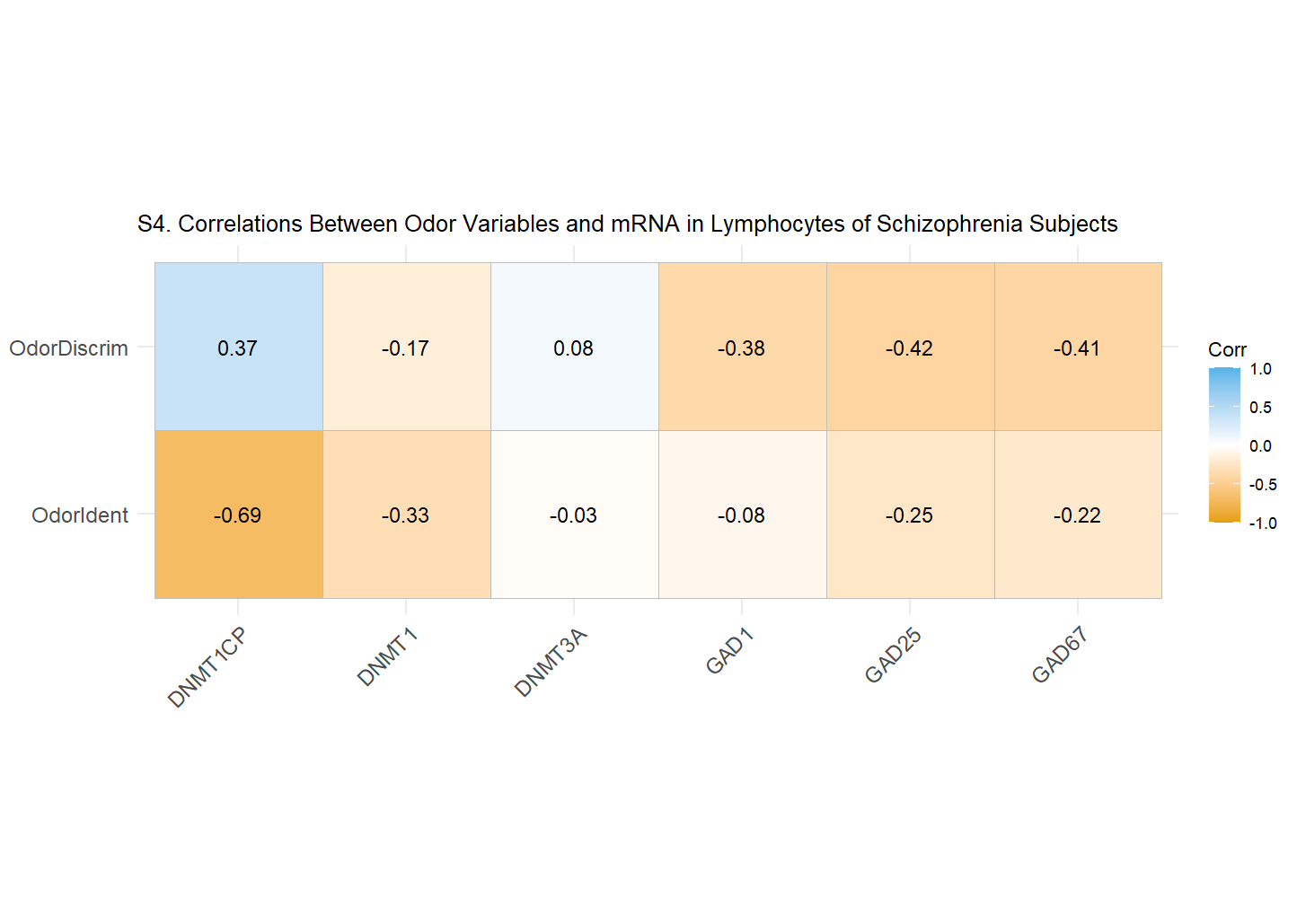

Supplement: Supplementary file 1 [file Data_Sheet_1.docx]
